# Pore and Thermochemical Properties of Biochar Materials Produced from Moso Bamboo Under Different Carbonization Conditions

**DOI:** 10.3390/ma19020310

**Published:** 2026-01-13

**Authors:** Hervan Marion Morgan, An-De Yan, Yong-Shun Lu, Chi-Hung Tsai, Wen-Tien Tsai

**Affiliations:** 1 Department of Tropical Agriculture and International Cooperation, National Pingtung University of Science and Technology, Neipu Township, Pingtung 912, Taiwan; p11222364@mail.npust.edu.tw; 2 Department of Forestry, National Pingtung University of Science and Technology, Neipu Township, Pingtung 912, Taiwan; dora50716059@gmail.com (A.-D.Y.); sern127348@gmail.com (Y.-S.L.); 3 Department of Resources Engineering, National Cheng Kung University, Tainan 701, Taiwan; n48091089@gs.ncku.edu.tw; 4 Graduate Institute of Bioresources, National Pingtung University of Science and Technology, Neipu Township, Pingtung 912, Taiwan

**Keywords:** moso bamboo, thermochemical property, biochar, pore property, elemental analysis

## Abstract

In this study, moso bamboo (*Phyllostachys edulis*, PE) was pyrolyzed in a high-temperature carbonization furnace to produce porous biochar materials with high carbon contents under different carbonization temperatures (500, 600, 700, 800, and 900 °C) and heating rates (10 and 20 °C/min). Preliminary characterization of the PE precursor was conducted to evaluate its thermochemical properties, including proximate analysis, elemental analysis, and thermal decomposition behavior. The results indicated that PE biomass is a suitable precursor for biochar production at temperatures above 400 °C, owing to its low ash content (<1%) and high volatile matter (>80%). The pore structure and thermochemical properties of PE-derived biochars were found to improve with increasing carbonization temperature. Optimal pore characteristics were achieved at 800 °C with a heating rate of 10 °C/min, resulting in a Brunauer–Emmett–Teller (BET) surface area of 496 m^2^/g and a total pore volume of 0.18 cm^3^/g. In contrast, biochars produced at a heating rate of 20 °C/min exhibited significantly higher carbon contents (90.7–95.7%) compared with those obtained at 10 °C/min (75.4–89.0%). This phenomenon was attributed to enhanced carbon volatilization associated with the longer residence time during slower heating. Observations from scanning electron microscopy (SEM) were consistent with the development of porous structures in the PE-based biochars.

## 1. Introduction

Bamboo is a fast-growing, strong, and perennial woody grass belonging to the Poaceae family rather than a tree, reaching maturity within approximately 3–5 years. It is widely distributed across tropical, subtropical, and mild temperate regions, particularly in Asia. In Taiwan, approximately 90 bamboo species are cultivated over an area exceeding 170,000 hectares [1]. Among these, the six most economically important species include ma bamboo, moso bamboo (*Phyllostachys edulis*), thorny bamboo (*Bambusa stenostachya*), Oldham’s bamboo, long-branch bamboo (*Bambusa dolichoclada*), and the endemic Makino bamboo (*Phyllostachys makinoi*). Notably, moso bamboo, which is native to Taiwan, is a giant species that has been extensively utilized in the textile industry and increasingly adopted as a green building material.

Bamboo is characterized by hollow culms with distinct nodes, high tensile strength, renewability as a lignocellulosic resource, and inherent antibacterial properties. These attributes have enabled its widespread application in eco-friendly products, including construction materials, textiles, charcoal materials, and other bamboo-based products [2,3,4,5,6,7,8]. Owing to its high content of lignocellulosic components namely cellulose, hemicellulose, and lignin, bamboo and its derived carbon materials (e.g., charcoal, biochar, and activated carbon) have been widely applied as energy sources and as adsorbents for the removal of environmental pollutants from air and water systems [9,10,11,12,13,14]. More importantly, bamboo has been recognized as a nature-based solution (NbS) for climate change mitigation and adaptation, due to its substantial carbon sequestration capacity, long-term carbon storage in durable bamboo products that substitute for high-emission materials such as steel and plastics, and its additional environmental benefits related to soil stabilization and bioenergy production [15,16]. Given its high lignocellulosic content and low ash content, bamboo has been regarded as an excellent precursor for the production of porous carbon materials (i.e., charcoal or biochar), with increasing attention paid to their value-added applications in recent studies [12,13,17,18,19].

For the production of biomass-based charcoal (commonly referred to as biochar), lignocellulose-rich precursors must be thermally treated under oxygen-limited conditions to prevent complete combustion and excessive ash formation. During thermal decomposition (carbonization), pyrolysis enhances the carbon content while reducing oxygen-containing functional groups, thereby generating liquid (bio-oil), gaseous products, and a solid biochar with a porous structure [20]. The yields and compositions of these products are primarily influenced by the feedstock type, heating rate, carbonization temperature, and residence time [21,22,23,24,25]. In addition to these primary parameters, several operational factors have been widely reported to significantly affect the physicochemical properties of biochars. These include surface mass loading, particle size, reactor configuration, gas flow conditions, and heat and mass transfer limitations within the biomass bed. Residence time, in particular, governs the extent of secondary devolatilization reactions, carbon matrix rearrangement, and aromatization, thereby influencing biochar yield, surface functionality, and structural stability. Similarly, surface mass loading affects local temperature gradients and volatile diffusion pathways, which may alter pore development, ash distribution, and the degree of carbon burn-off under otherwise identical thermal conditions. Although these parameters are recognized as critical in determining biochar characteristics, their systematic investigation was beyond the scope of the present study. Accordingly, the experimental design was deliberately constrained to isolate the effects of carbonization temperature and heating rate under a fixed residence time and biomass loading, enabling a clearer interpretation of their individual contributions to biochar formation. With respect to the production of biochar from moso bamboo (*Phyllostachys edulis*, also known as *Phyllostachys pubescens*), only a limited number of studies have been reported in recent years [26,27,28,29].

Huang et al. [26] investigated the production of charcoal at temperatures ranging from 600 to 1000 °C with a holding time of 120 min and heating rates of 5–10 °C/min, demonstrating that the Brunauer–Emmett–Teller (BET) surface area increased with carbonization temperature, reaching approximately 90 m^2^/g at 600 °C, 120 m^2^/g at 800 °C, and 150 m^2^/g at 1000 °C. Chen et al. [27] produced biochars at temperatures between 300 and 700 °C using a fixed heating rate of 10 °C/min. As a result of enhanced thermal decomposition at elevated temperatures, the biochar yield decreased from 49.3% at 300 °C to 23.6% at 700 °C, while the carbon content and BET surface area increased from 68.6% and 12.3 m^2^/g to 88.3% and 64.5 m^2^/g, respectively. Similarly, Wang et al. [28] conducted pyrolysis experiments at temperatures of 400–600 °C with a heating rate of 10 °C min^−1^, reporting a sharp increase in BET surface area from 2.2 m^2^/g at 400 °C and 6.5 m^2^/g at 500 °C to 181.1 m^2^/g at 600 °C. Liu et al. [29] produced charcoal at 650 °C with a holding time of 2 h and subsequently modified the material using sodium hydroxide (NaOH) and ferrous/ferric chloride (FeCl_2_/FeCl_3_) impregnation. The modified biochars exhibited significantly higher BET surface areas (63.0 and 84.7 m^2^/g for NaOH-impregnated and magnetic biochars, respectively) compared with the unmodified biochar (1.4 m^2^/g).

In the previous study [30], an induction-heating pyrolysis system was employed to produce porous bamboo-derived biochars at high heating rates (110–170 °C/min). The results indicated that a heating rate of approximately 150 °C/min was optimal for generating microporous biochars with BET surface areas exceeding 250 m^2^/g and pore volumes greater than 0.12 cm^3^/g. In contrast to the predominantly high heating-rate conditions reported in the literature, the present study systematically examines the effects of low heating-rate carbonization across a wide temperature range, with simultaneous evaluation of pore structure evolution and elemental composition, thereby providing complementary mechanistic insight into bamboo biochar formation. To further elucidate the effects of carbonization temperature and heating rate on pore development and elemental composition, pyrolysis experiments were conducted in the present study at temperatures of 500, 600, 700, 800, and 900 °C using heating rates of 10 and 20 °C/min with a holding time of 30 min. The pore properties, textural structures, and elemental compositions of the resulting biochars were systematically characterized using nitrogen adsorption–desorption isotherms, scanning electron microscopy (SEM), and elemental analysis. By comparison with the properties of the original moso bamboo precursor, the influences of processing conditions on biochar characteristics were comprehensively evaluated.

## 2. Materials and Methods

### 2.1. Moso Bamboo

**Figure 1:**
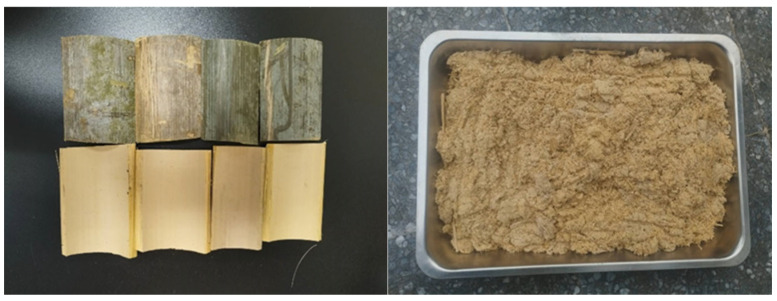
Moso bamboo used in this work (Left: sliced plate, right: shredded filament).

The starting material, moso bamboo (denoted as PE), was obtained from a local plantation in Zhushan Township, Nantou County, Taiwan. As illustrated in Figure 1, the bamboo culms with hollow cylindrical structures were first cut into thin slices and subsequently shredded into filamentous particles. The resulting filaments were sieved to obtain particle sizes ranging from 1.70 mm (mesh No. 12) to 0.841 mm (mesh No. 20), with an average particle size of approximately 1.27 mm. The sieved bamboo filaments were then dried in an oven at 105 °C overnight to remove residual moisture. Consequently, all thermochemical characteristics of the moso bamboo were determined on a dry basis. To minimize moisture uptake prior to analysis, the dried samples were stored in an oven maintained at 60 °C until thermochemical characterization and carbonization experiments were conducted.

### 2.2. Determinations of Thermochemical Characteristics of Moso Bamboo

As The thermochemical characteristics of moso bamboo were determined following the procedures described in previous studies [31,32]. Thermogravimetric analysis (TGA) and derivative thermogravimetric (DTG) analysis were performed using a TGA-51 analyzer (Shimadzu Co., Tokyo, Japan). Measurements were conducted over a temperature range of 25–900 °C at heating rates of 5, 10, 15, and 20 °C/min under a continuous nitrogen purge to maintain an inert atmosphere. Proximate analysis was carried out to determine the contents of ash, volatile matter, and fixed carbon (calculated by difference). The higher heating value (HHV) was measured using a bomb calorimeter (CALORIMETER ASSY 6200, Parr Co., Moline, IL, USA). Baseline elemental compositions of moso bamboo were determined using a vario EL cube elemental analyzer (Elementar GmbH, Langenselbold, Germany), which enabled automated, high-precision determination of total carbon (as CO_2_), hydrogen (as H_2_ or H_2_O), nitrogen (as N_2_), oxygen (as CO), and sulfur (as SO_2_) [33].

### 2.3. Carbonization Experiments

Carbonization experiments were conducted in a vertical furnace following the procedures described in previous studies [31,32]. The bamboo samples were heated from room temperature to final temperatures of 500, 600, 700, 800, and 900 °C, at heating rates of 10 and 20 °C/min. An inert atmosphere was maintained throughout the experiments by continuously supplying nitrogen gas at a flow rate of 500 cm^3^/min. For each experiment, the final temperature was maintained for 30 min to ensure sufficient carbonization. The yields of the resulting carbon materials (biochar products) were calculated by measuring the mass difference between the initial bamboo samples (approximately 5 g) and the obtained biochars. Prior to pore property and elemental analyses, the solid biochar samples designated as PE-temperature-rate, were dried and stored in an air-circulating oven at 105 °C. For example, PE800-10 denotes the biochar produced from moso bamboo (PE) carbonized at 800 °C using a heating rate of 10 °C/min.

### 2.4. Determinations of Pore and Thermochemical Characteristics of PE-Based Biochar Materials

The pore and thermochemical characteristics of the PE-based biochar products were analyzed using multiple analytical instruments, including an automatic ASAP 2020 Plus adsorption–desorption analyzer (Micromeritics Co., Norcross, GA, USA), an elemental analyzer as described in Section 2.2, and a scanning electron microscope (S-3000N, Hitachi Co., Tokyo, Japan). For pore structure analysis, nitrogen adsorption–desorption isotherms were measured at −196 °C. The Brunauer–Emmett–Teller (BET) surface area was calculated from adsorption data in the relative pressure (P/P_0_) range of 0.05–0.30, yielding high correlation coefficients (>0.995) [34,35]. The total pore volume was determined from the amount of nitrogen adsorbed at high relative pressure (P/P_0_ > 0.99), with the adsorbed gas volume converted to the corresponding liquid volume based on nitrogen density [35]. Pore size distribution was evaluated using the Barrett–Joyner–Halenda (BJH) method, with particular emphasis on mesopores with diameters ranging from 2 to 50 nm [35]. In addition, the surface morphologies and porous structures of the raw moso bamboo and selected PE-based biochar samples exhibiting higher pore properties were examined using scanning electron microscopy.

## 3. Results and Discussion

### 3.1. Thermochemical Characteristics of Moso Bamboo

As summarized in Table 1, moso bamboo exhibited high carbon and volatile matter contents of 52.15 wt.% and 81.83 wt.% (dry basis), respectively, along with low sulfur (0.08 wt.%) and ash contents (0.91 wt.%). By definition, the fixed carbon content was calculated to be approximately 17.23 wt.%, representing the non-volatile carbon fraction remaining in the solid char after devolatilization. These results were in good agreement with previously reported values in the literature [14,27,28]. Consequently, the higher heating value of moso bamboo was determined to be 20.35 MJ/kg. The elemental composition of moso bamboo, as also presented in Table 1, was characterized by high contents of carbon (52.15 wt.%), hydrogen (6.19 wt.%), and oxygen (41.26 wt.%). Based on molar ratios, the empirical chemical formula of moso bamboo was estimated as C_4.3_H_6.2_O_2.6_, which closely resembles that of typical lignocellulosic biomass. On the basis of these thermochemical properties, moso bamboo was confirmed to be a suitable precursor for biochar production via pyrolysis [36].

**Figure 2:**
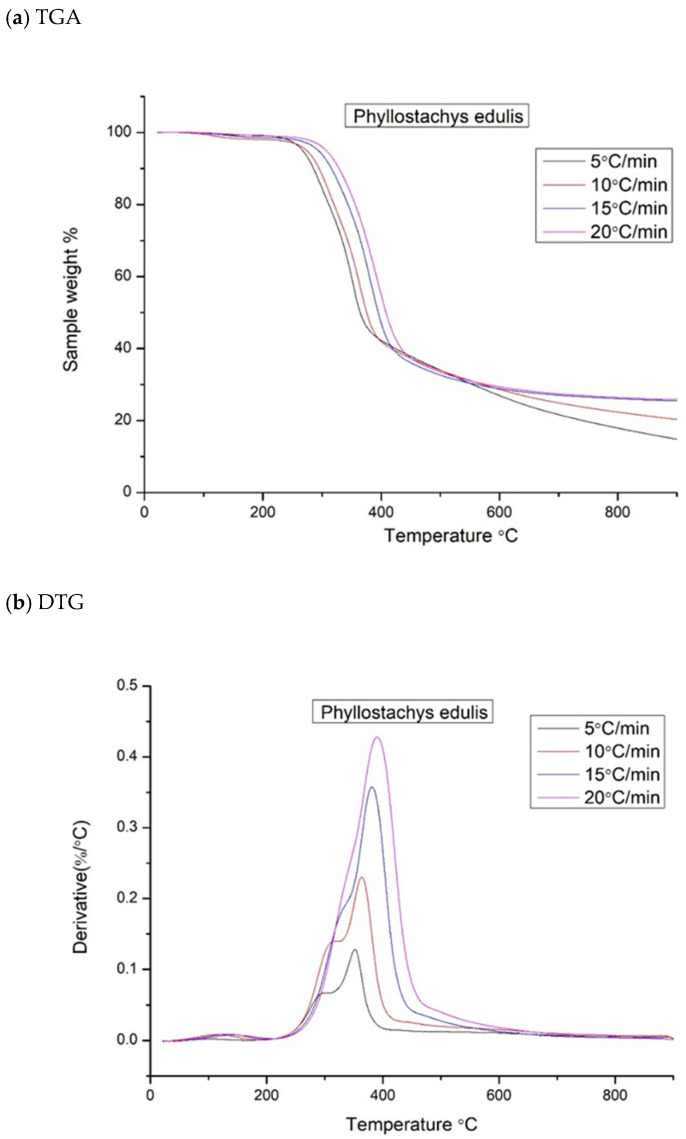
(**a**) Thermogravimetric analysis curves of moso bamboo and (**b**) its derivative thermogravimetry (TGA/DTG) curves at the heating rates of 5, 10, 15 and 20 °C/min.

To elucidate the thermal decomposition behavior of moso bamboo during carbonization, thermogravimetric (TGA) and derivative thermogravimetric (DTG) analyses were performed at heating rates of 5, 10, 15, and 20 °C/min, as shown in Figure 2. The TGA/DTG profiles exhibited similar decomposition patterns, with a progressive shift toward higher temperatures as the heating rate increased [37,38]. This behavior was attributed to thermal lag effects, whereby higher heating rates require additional energy and time for heat transfer within the non-conductive lignocellulosic biomass, resulting in delayed decomposition and higher apparent decomposition temperatures [39]. The thermal decomposition of moso bamboo was observed to proceed through three distinct stages. Stage I (50–200 °C) corresponded to the evaporation of free moisture and the release of light volatile compounds, accounting for approximately 2 wt.% mass loss. Stage II (200–450 °C) represented the primary devolatilization region, during which about 60 wt.% mass loss occurred due to the thermal decomposition of lignocellulosic components, particularly hemicellulose. DTG analysis indicates that Stage II proceeds through a complex, multi-step mechanism characterized by overlapping degradation reactions of hemicellulose and cellulose. The thermal decomposition of hemicellulose is generally initiated at lower temperatures (≈200–300 °C) and is associated with the cleavage of relatively weak acetyl, ether, and glycosidic bonds, resulting in the rapid evolution of CO_2_, CO, acetic acid, and other oxygenated volatile species. With further temperature increase (≈300–400 °C), cellulose is subjected to depolymerization via the breakdown of β-1,4-glycosidic linkages [39], leading to the formation of levoglucosan, light hydrocarbons, and condensable tar compounds. The overlap of these degradation processes gives rise to the broadened and asymmetric DTG peak observed in Figure 2b, reflecting the concurrent occurrence of intense devolatilization, secondary cracking of volatile products, and the initial formation of char. The pronounced mass loss and elevated DTG peak intensity during this stage highlighted its dominant role in governing char yield, pore development, and surface chemical characteristics. Stage III (450–900 °C) involved continuous and gradual devolatilization (approximately 30 wt.%), mainly associated with lignin degradation and further carbon matrix rearrangement. In the present study, carbonization temperatures above 500 °C were selected to ensure sufficient charring and the development of porous carbon structures. However, it was noted that higher carbonization temperatures inevitably resulted in lower solid yields due to intensified thermal decomposition reactions.

### 3.2. Pore Properties of PE-Based Biochar Materials

In this study, the pore properties of the PE-based biochar materials were evaluated in terms of Brunauer–Emmett–Teller (BET) surface area and total pore volume, as summarized in Table 2. The yields of the resulting biochar products obtained at heating rates of 10 and 20 °C/min were also reported in Table 2. In addition, the pore size distributions of biochar samples exhibiting higher BET surface areas were further analyzed using nitrogen adsorption–desorption isotherms measured at −196 °C. The effects of carbonization temperature and heating rate on pore development are discussed as follows.

**Figure 3:**
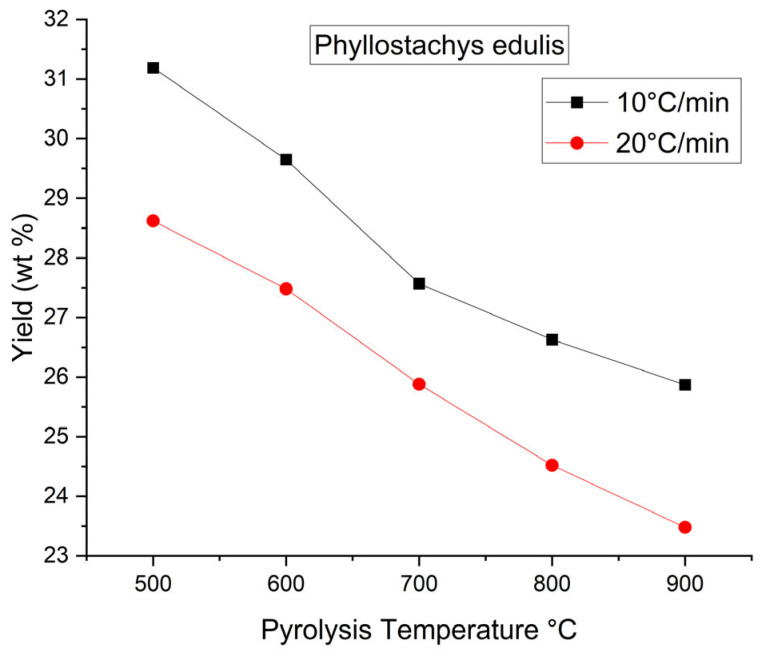
Biochar yield as a result of pyrolysis temperature at the different heating reates (10 and 20 °C/min).

**Figure 4:**
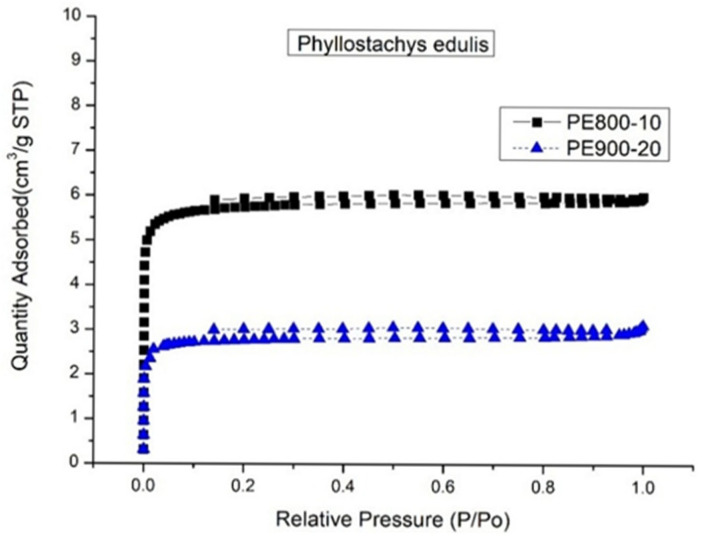
N_2_ adsorption–desorption isotherms of optimal biochar products (i.e., PE800-10 and PE900-20).

**Figure 5:**
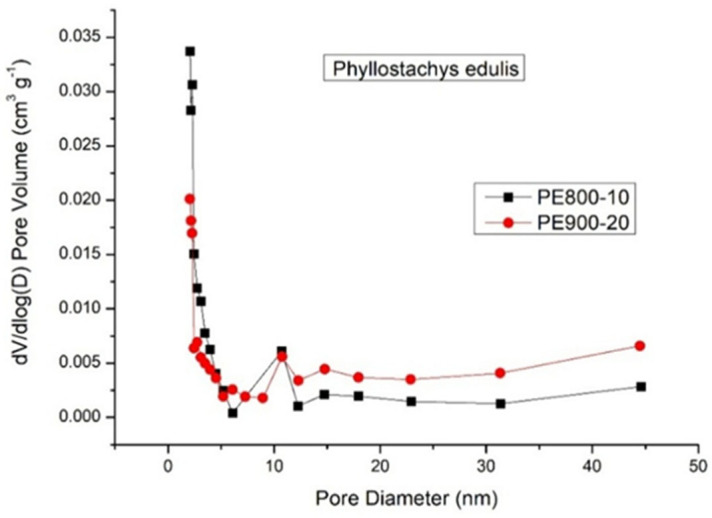
Pore size distributions of optimal plant carbon products (i.e., PE800-10 and PE900-20).

**Figure 6:**
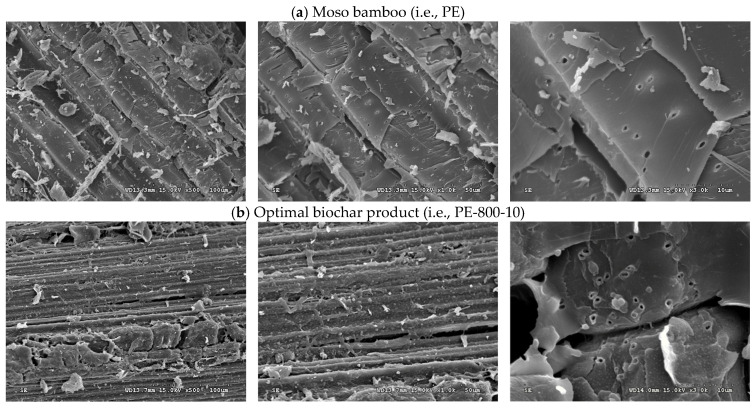
SEM images of (**a**) Moso bamboo (Left: ×500; Center: ×1000; Right: ×3000) and (**b**) Optimal biochar product (Left: ×500; Center: ×1000; Right: ×3000) (i.e., PE-800-10).

1. First, the biochar yield was observed to decrease with increasing carbonization temperature from 500 to 900 °C at both heating rates of 10 and 20 °C/min. This trend was attributed to enhanced thermal decomposition of the biomass, leading to increased gas evolution and a corresponding reduction in solid biochar yield. At higher carbonization temperatures, primary devolatilization reactions were intensified, leading to the extensive release of non-condensable gaseous products such as CO_2_, CO or CH_4_, and condensable tar species that were generated from the breakdown of residual lignocellulosic structures. Furthermore, secondary cracking reactions of tar intermediates were promoted at elevated temperatures, whereby condensable products were further converted into lighter gaseous compounds, thereby reducing the retained solid fraction. Concurrently, the carbon matrix rearrangement occurred through aromatization and ring condensation in the carbon framework. These processes may have been accompanied by the continuous elimination of heteroatoms in the form of volatile species which contributed to additional mass loss despite the formation of a more thermally stable carbon structure. As shown in Table 2, the reduction in yield between 500 and 900 °C was relatively moderate, which was consistent with the mass loss behavior observed in the TGA curves (Figure 2). This observation suggested that the majority of the readily volatilizable components were removed during the primary devolatilization stage, while subsequent yield reduction at higher temperatures was mainly governed by slower secondary devolatilization processes and carbon matrix restructuring. The visual representation of the relationship biochar yield had with pyrolysis temperature is presented in Figure 3.
2. Second, the BET surface area of the biochars produced at a heating rate of 10 °C/min increased sharply from 5.04 m^2^/g at 500 °C to 496.03 m^2^/g at 800 °C [40], followed by a slight decrease to 450.02 m^2^/g at 900 °C. The pronounced increase in BET surface area observed between 500 and 800 °C may be attributed to progressive pore formation and pore opening processes driven by intensified devolatilization reactions. As the carbonization temperature increased, the release of volatile compounds and tar intermediates from the lignocellulosic matrix generated internal voids, thereby promoting the development of micropores and small mesopores. Simultaneously, thermal reorganization of the carbon framework occurred through aromatization and structural condensation, which contributed to the stabilization and widening of newly formed pores. At 900 °C, the slight decline in surface area suggests that pore collapse and excessive burn-off became increasingly significant. Under such severe thermal conditions, continued carbon matrix rearrangement and ring condensation may have caused pore wall shrinkage, coalescence, or partial structural collapse. In addition, the accelerated removal of carbon atoms through gasification reactions likely reduced the integrity of thin pore walls, resulting in a net loss of accessible porosity despite increased carbon ordering [41]. In contrast, biochars produced at a heating rate of 20 °C/min exhibited a pronounced increase in BET surface area only at elevated temperatures, rising from 1.96 m^2^/g at 800 °C to 240.04 m^2^/g at 900 °C, with minimal pore development observed in the temperature range of 500–800 °C. This behavior may be explained by kinetic limitations associated with rapid heating, whereby insufficient residence time was available for gradual volatile release and controlled pore development at lower temperatures. As a result, pore formation was suppressed until higher temperatures were reached, at which point intensified devolatilization and structural rearrangement processes facilitated delayed pore opening. Overall, the biochar with the most developed pore structure characterized by a BET surface area of 496.03 m^2^/g and a total pore volume of 0.1771 cm^3^/g was obtained at 800 °C using a heating rate of 10 °C/min with a holding time of 30 min. This condition appears to represent an optimal balance between volatile-driven pore generation, carbon matrix reorganization, and structural stabilization, prior to the onset of significant pore collapse or burn-off at higher temperatures.
3. Third, the nitrogen adsorption–desorption isotherms of the biochar samples exhibiting optimal pore properties, namely PE800-10 and PE900-20, are presented in Figure 4. The presence of a distinct “knee” at low relative pressures indicated Type I isotherm behavior, which is characteristic of microporous carbon materials with pore diameters smaller than 2.0 nm [34,35]. In addition, a pronounced hysteresis loop (Type IV isotherm behavior) was observed in the relative pressure (P/P_0_) range of 0.2–0.9, suggesting the occurrence of capillary condensation within mesopores having diameters between 2.0 and 50.0 nm. Based on analysis of the adsorption–desorption data using the Barrett–Joyner–Halenda (BJH) method [35], the corresponding pore size distribution curves for PE800-10 and PE900-20 were derived and are shown in Figure 5. Both samples exhibited a dominant mesopore size centered at approximately 11 nm.
4. Finally, scanning electron microscopy (SEM) observations revealed a marked difference in surface morphology between the raw moso bamboo (PE) and the optimized biochar product (PE800-10). As shown in Figure 6, the pristine bamboo exhibited a relatively smooth and weakly porous surface, whereas the PE800-10 biochar displayed a highly developed porous structure, consistent with its significantly enhanced BET surface area and pore volume.

### 3.3. Elemental Analysis of PE-Based Biochar Materials

The elemental compositions of the PE-based biochar materials were determined using an elemental analyzer (EA) based on complete combustion followed by gas chromatographic analysis of the resulting gases, including CO_2_, H_2_O, H_2_, N_2_, and SO_2_. The obtained elemental contents (wt.%) are summarized in Table 3, illustrating the variations in carbon, hydrogen, and oxygen contents as functions of carbonization temperature (500–900 °C) and heating rate (10–20 °C/min).

At a heating rate of 10 °C/min, the carbon content of the resulting biochars increased from 75.44 wt.% at 500 °C to 88.99 wt.% at 800 °C, followed by a slight decrease to 87.26 wt.% at 900 °C. A similar trend was observed for biochars produced at a heating rate of 20 °C/min, for which the carbon content increased from 90.74 wt.% at 500 °C to a maximum of 95.61 wt.% at 800 °C, before decreasing to 92.36 wt.% at 900 °C. In contrast, the hydrogen and oxygen contents generally decreased with increasing carbonization temperature for both heating rates.

The progressive increase in carbon content accompanied by the reduction in hydrogen and oxygen contents may be attributed to temperature-driven dehydrogenation and deoxygenation reactions that occurred during pyrolysis. As carbonization temperature increased, hydrogen was preferentially removed in the form of H_2_ and H_2_O through dehydration, decarboxylation, and cracking reactions, while oxygen was eliminated primarily as CO and CO_2_ via decarboxylation and decarbonylation pathways. These reactions led to a continuous decrease in the H/C and O/C atomic ratios, indicating a gradual transition from aliphatic structures toward more condensed carbon frameworks. Concurrently, aromatization processes were promoted at elevated temperatures through structural rearrangement, ring condensation, and the fusion of polyaromatic domains. The removal of heteroatoms facilitated closer packing of carbon layers and increased structural ordering, resulting in the formation of thermally stable, aromatic-rich biochar matrices. This evolution is consistent with the observed enrichment of carbon content up to 800 °C and reflects the dominance of carbon condensation reactions over volatilization processes within this temperature range. The slight decrease in carbon content and the concurrent increase in oxygen content observed at 900 °C for both heating rates may be attributed to enhanced carbon burn-off and surface re-oxidation effects. Under severe carbonization conditions, partial gasification of the carbon matrix may occur, leading to the loss of carbon as gaseous species. In addition, the formation of thermodynamically stable oxygen-containing surface functional groups or post-carbonization exposure to residual oxygen may contribute to the apparent increase in oxygen content at the highest temperature.

Overall, these compositional changes were associated with intensified cracking reactions, volatile release, and progressive carbon structure condensation at elevated temperatures, leading to the formation of increasingly aromatic and carbon-rich biochar materials. To further elucidate the relative contributions of dehydrogenation, deoxygenation, and aromatization processes, complementary analysis of evolved gaseous products (e.g., H_2_, H_2_O, CO, and CO_2_) at higher carbonization temperatures (800–900 °C) using gas chromatography or Fourier transform infrared spectroscopy (FTIR) would be beneficial, as reported in previous studies [27].

## 4. Conclusions

Based on the thermochemical characteristics determined in this study, moso bamboo (PE) was demonstrated to be a suitable precursor for the production of porous carbon materials under appropriate pyrolysis conditions. The effects of carbonization temperature (500, 600, 700, 800, and 900 °C, with a holding time of 30 min) and heating rate (10 and 20 °C/min) on the pore properties and elemental compositions of biochars derived from moso bamboo, a renewable and fast-growing lignocellulosic biomass were systematically investigated. The results indicated that both carbonization temperature and heating rate exerted significant influences on the physicochemical properties of the resulting biochars. Although a relatively high BET surface area of 240.04 m^2^/g was achieved at 900 °C using a heating rate of 20 °C/min, the most developed pore structure characterized by a BET surface area of 496.03 m^2^/g and a total pore volume of 0.1771 cm^3^/g was obtained at a carbonization temperature of 800 °C with a heating rate of 10 °C/min. These trends suggest the existence of an optimal carbonization temperature window in which volatile release promotes effective pore opening and structural development, while excessive carbon matrix shrinkage and rearrangement at higher temperatures may compromise pore stability. However, an inverse relationship between biochar yield and carbonization severity was observed. Specifically, the biochar yield decreased from 31.19% at 500 °C to 25.87% at 900 °C at a heating rate of 10 °C/min, and from 28.62% to 23.38% over the same temperature range at 20 °C/min. This behavior may be attributed to the more gradual devolatilization and extended thermal exposure associated with lower heating rates, which facilitate carbon matrix reorganization and the stabilization of newly formed pores. Elemental analysis revealed an overall increase in carbon content and a corresponding decrease in hydrogen and oxygen contents with increasing temperature, reflecting progressive dehydrogenation, deoxygenation, and aromatization during carbonization. The evolution of elemental composition provides mechanistic evidence of the transformation from aliphatic biomass components to condensed aromatic carbon structures, which is closely associated with improved thermal stability and altered surface chemistry of the biochars. Overall, the results demonstrate that careful control of carbonization temperature and heating rate is critical for tailoring the structural and chemical properties of bamboo-derived biochars. The insights gained from this study contribute to a deeper understanding of structure–process relationships under slow heating-rate conditions and provide guidance for optimizing biochar production for adsorption and other environmental applications.

## Figures and Tables

**Table 1:** Proximate analysis and calorific value of moso bamboo.

Property ^a^	Value
Proximate analysis ^a,c^	
Ash (wt.%)	0.91 ± 0.1
Volatile matter (wt.%)	81.83 ± 0.95
Fixed carbon ^b^ (wt.%)	17.26
Calorific value ^a,c^ (MJ/kg)	20.35
Elemental analysis ^c^	
Carbon (wt.%)	52.15
Oxygen (wt.%) ^b^	41.26
Hydrogen (wt.%)	6.19
Nitrogen (wt.%)	0.31
Sulfur (wt.%)	0.08

^a^ The mean ± standard deviation for three determinations. ^b^ By difference. ^c^ On a dry basis.

**Table 2:** Values of yield, BET surface area total pore volume for PE-based biochar materials.

PE-Based Biochar ^a^	Yield (wt.%)	S_BET_^b^ (m^2^/g)	V_t_^c^ (cm^3^/g)
PE500-10	31.19	5.04	0.0017
PE600-10	29.65	94.31	0.0552
PE700-10	27.57	55.82	0.0168
PE800-10	26.63	496.03	0.1771
PE900-10	25.87	450.02	0.1602
PE500-20	28.62	1.98	0.0025
PE600-20	27.48	1.25	0.0035
PE700-20	25.88	0.74	0.0008
PE800-20	24.52	1.96	0.0020
PE900-20	23.48	240.04	0.0852

^a^ Sample notation indicated the resulting biochar, produced at the pyrolysis temperatures of 400–900 °C and heating rates of 10–20 °C/min for a holding time of 30 min using 5 g PE. ^b^ BET surface area (S_BET_) was calculated from relative pressure range of 0.05–0.30 (15 points). ^c^ Total pore volume (Vt) obtained at relative pressure of about 0.995.

**Table 3:** Elemental compositions of PE-based biochar materials.

Biochar	C (wt.%)	O (wt.%)	H (wt.%)	N (wt.%)	S (wt.%)	H/C Ratio	O/C Ratio
PE500-10	75.44	20.96	2.81	0.64	0.15	0.037	0.278
PE600-10	85.47	11.27	2.54	0.61	0.12	0.029	0.132
PE700-10	86.75	10.73	1.88	0.55	0.10	0.022	0.124
PE800-10	88.99	8.92	1.50	0.50	0.10	0.017	0.100
PE900-10	87.26	10.69	1.43	0.51	0.12	0.016	0.125
PE500-20	90.74	5.08	3.29	0.59	0.08	0.036	0.056
PE600-20	91.34	5.51	2.52	0.56	0.07	0.028	0.060
PE700-20	95.73	1.65	1.87	0.68	0.08	0.019	0.017
PE800-20	95.61	2.21	1.54	0.57	0.07	0.016	0.023
PE900-20	92.36	5.69	1.28	0.58	0.09	0.014	0.062

## Data Availability

The original contributions presented in this study are included in the article. Further inquiries can be directed to the corresponding authors.
